# Crisis of Authority: The Truth of Post-Truth

**DOI:** 10.1007/s10767-021-09415-6

**Published:** 2021-10-21

**Authors:** Henrik Enroth

**Affiliations:** Department of Political Science, Linnaeus University, 351 95 Vaxjo, Sweden

**Keywords:** Post-truth, Authority, Crisis, Hannah Arendt, Populism

## Abstract

This article is a critique of the notion of post-truth. Drawing on the work of Hannah Arendt, I argue that the epistemological crisis suggested by the notion of post-truth is epiphenomenal to a more general crisis of authority, a crisis that is poorly understood in the literature. I also argue that revisiting Arendt’s account of authority can help us elucidate the vexed dynamics of authority in modern society, as well as the dynamics behind its current crisis. The post-truth situation is a loss of authority that is political before it presents as epistemological. Effectively addressing this situation, I conclude, is a much more challenging and complex proposition than what is suggested in the literature on post-truth.

Whenever the prefix “post” insinuates itself into our thinking, there is good reason to think again. This ubiquitous latinism is conspicuously void of information. “Post” simply suggests that something is following upon, and supposedly surpassing, something else, without telling us anything specific about the nature of what follows, or the nature of what it follows upon, or how we got from the one to the other. “Postmodernism” is a famous case in point (cf. Foucault, 1984: 39). A more recent case in point is the notion that these are “post-truth” or “post-factual” times (see, e.g., d’Ancona, 2017; Block, 2019; Hyvönen, 2018; Kalpokas, 2019; McIntyre, 2018; Newman, 2019; Sim, 2019). In the deluge of academic, journalistic, and hybrid writing that has brought this notion to the fore, it has been suggested that we are in the midst of a “new war on truth” (d’Ancona, 2017), a metaphor that conveys a pervasive attitude in this literature, and also its default trope, which is not metaphor but hyperbole. Among the notable casualties in the new war we find, in addition to “truth,” “facts,” and “reason,” also “the Enlightenment,” which is now, we are told, “really dead,” since the Enlightenment “displaced the primacy of myths with hard facts” and hard facts have now in turn been displaced by “arguments based on their emotional appeal and symbolic value and subjective rather than impersonal truth” (Kalpokas, 2019: 2).

This article is a critique of the notion of post-truth, and a reconsideration of the situation to which this notion inadequately refers. Drawing on the work of Hannah Arendt, I argue that the epistemological crisis suggested by the misnomer “post-truth” is epiphenomenal to a more general crisis of authority, the nature of which has so far escaped the post-truth literature. Revisiting Arendt’s account of authority can help us elucidate both the general dynamics of authority in a modern democracy and the specific dynamics behind its current crisis. My main errand in this piece is thus to change perspectives on post-truth, hopefully for the benefit of our understanding of what is ailing us. This amounts to a look at what I take to be the big picture from which the purported post-truth situation is a cut-out and a blow-up. What follows is a zooming-out from the epistemological notions that dominate the literature and a proposal to approach the post-truth situation differently, analytically as well as politically.

The Enlightenment and its supposed death aside, the post-truth literature takes its point of departure in 2016. This was the year when the *Oxford English Dictionary* picked “post-truth” as its “Word of the Year,” and offered the following by way of explication: “Originally *U.S.* Relating to or denoting circumstances in which objective facts are less influential in shaping political debate or public opinion than appeals to emotion and personal belief” (OED). With all due respect to the *Oxford English Dictionary*, whose job, in this case, is to document the muddled thinking of the moment, this definition does not capture, as suggested, the malaise of our times as much as the need to think again about the malaise of our times. Insofar as the term refers to something beyond hyperbole and sheer hype, “post-truth” is, I suggest, a surface phenomenon, a symptom, not the ailment. Empirically, this notion hints at some of the most monumental and disturbing challenges facing us at this moment, most notably climate change denial, illiberal populism, and the resurgence of authoritarianism. Conceptually and theoretically, however, the notion of post-truth does not give us the resources we need to understand and analyze these phenomena, nor, in consequence, the resources we need to counteract them.

The fact — I continue to rely on this untimely word — that 2016 was the year when the *Oxford English Dictionary* picked “post-truth” as its Word of the Year suggests the extent to which this notion is not just “originally U.S.,” but inextricably tied to the presidency, and to the person, of Donald J. Trump, whose famously flexible relationship to truth is well captured by the entry in the *OED*. Unsurprisingly, Trump and the Trump presidency figure prominently in the literature on the subject. Yet, for all the damage they have done, neither Trump nor his enablers have put the Enlightenment to rest, and nothing is gained by reducing our predicament to this traumatizing episode in the history of the USA, however grave the episode and its fallout. In sociological rather than characterological terms, the general situation or condition in which the post-truth phenomenon has manifested is not made legible as much as rendered obscure by this notion, and this is a pervasive condition, by no means restricted to the USA. It is also, arguably, a critical condition, but one for which “post-truth” is an inadequate label, and which the discourse on post-truth is of little help in diagnosing, let alone administering remedies for.

In this paper, my interest in Arendt’s work lies in its use-value in the present, not in what Arendt herself might have intended its use-value to be in the past. This bears pointing out not least since I make use of Arendt’s thoughts on authority more than of her now frequently quoted remarks on truth and politics, themes which she herself, interestingly, did not connect (Arendt, 1993a, b). To help us unpack the post-truth predicament, Arendt’s thoughts on authority have greater use-value, I contend, than her comments on the fraught relationship between truth and politics. And while the latter have been liberally referenced in the literature on post-truth, the potential of the former in this context remains to be explored.

A word on “crisis,” which is not a word I use lightly. As István Hont noted many years ago, this is a word that has long been “overused, generalized and trivialized” (1994: 167). In European history, prior to its introduction in modern political discourse, “crisis” did service as a medical term, designating “a moment of crucial decision in the face of acute difficulty or danger,” the “turning point,” as Hont has put it, “where a road to recovery had to be opened if an irrecoverable descent into oblivion was to be avoided” (1994: 167; cf. Koselleck, 1982). This etymologically original, medical-metaphorical sense of the term is the one I intend here, rather than the modern sociopolitical usage in which crisis and revolution became interrelated, so that “revolution was justified as a solution to a crisis and then seen as the very source of the next crisis” (Hont, 1994: 168; Koselleck, 1988). The solution to our present crisis, I argue, is a reconstitution of authority, which is ambitious and radical enough but nothing like revolution in the political sense. And if this is indeed the turning point where the road to recovery has to be opened, then the most crucial decision, before we can hope to arrive at a solution, is the diagnosis itself.

## Crisis of Epistemology?

For my purposes, the literature on post-truth can be roughly divided into two categories. First, there is what I refer to as the epistemological diagnosis of our situation. This literature has come to dominate both academic and public discourse, and contributions are frequently at the crossroads between them. In this literature, epistemological notions such as truth, facts, and reason are the focus of the diagnosis, and the main suggestion is that these are under attack on a new scale or in new and insidious ways. Societal, political, and cultural factors may enter the diagnosis as explanations for such attacks or their effects, but not as integral parts of the ailment itself (e.g., d’Ancona, 2017; McIntyre, 2018; Sim, 2019). Second, there is a growing body of politico-theoretically oriented literature in which notions of truth and facts are still in focus, but in which it is suggested that the post-truth condition is in itself genuinely societal, political, or cultural in nature, rather than solely or primarily epistemological (e.g., Block, 2019; Hyvönen, 2018; Kalpokas, 2019; Newman, 2019; Zerilli, 2019).

With regard to the latter literature, I am certainly not the first to enlist Arendt in response to the post-truth situation, nor am I the first to suggest that the nature of our crisis is political more than epistemological (Hyvönen, 2018; Newman, 2019; Zerilli, 2019). But the evidence so far presented and discussed in this literature of post-truth as a “crisis of the political” (Newman, 2019) is striking in its range and heterogeneity, including “the media environment” (Hyvönen, 2018: 45), a “neoliberal consensus” (Newman, 2019: 99), and, in the spirit and idiom of Arendt, a loss “not of truth as such but of a common world” (Zerilli, 2019: 157–158). Conceptually and theoretically, the political aspects of the post-truth situation remain elusive, and allusive, in the literature. To conceptualize and theorize this in a more exact fashion, I will work my way from a critical reading of the epistemological literature on post-truth to an analysis of post-truth as symptom of a crisis of authority, in a specifically Arendtian sense. As I hope to show, this will allow us to not only flesh out the loss of a common world, which is indeed an aspect of what we are witnessing, but also, importantly, to get a clearer view of what is involved in the making and remaking of a common world. This is what is at stake in Arendt’s ruminations on authority, and this is also what has so far been neglected or underemphasized in the discourse on post-truth.

My main target in this section and the next is the epistemologically oriented literature on post-truth. David Brooks captured the general sentiment in this literature when he announced, in an op-ed column in *The New York Times* in the aftermath of the 2020 US presidential election: “We live in a country in epistemological crisis” (Brooks, 2020). Cast in the language of epistemology, the crisis to which Brooks alludes comes across as vaguely Kuhnian, somewhat like a paradigm crisis. As is well known, any paradigm crisis starts with anomaly, something unexpected and, from within the current paradigm, inexplicable (Kuhn, 1962). In this case, as suggested by the entry in the *Oxford English Dictionary*, the anomaly concerns how political debate and public opinion now seem to be shaped, presumably in contrast with how they were previously shaped.

On the one hand, there appears to be increasing disbelief in what has conventionally been regarded as our most reliable forms of knowledge and sources of information, notably science, illustrated at the time of writing by denial and downplaying of anthropogenic climate change, the current corona virus pandemic, and the benefits of vaccines. On the other hand, there appears to be increasing belief in demonstrably unreliable forms of knowledge and sources of information, such as conspiracy theories spiraling the internet, or a president of the USA whom 27 psychiatrists and mental health experts and one estranged niece publicly declared incapable of distinguishing between truth and lie, between fact and self-serving fiction (Lee, 2017; Trump, 2020).

The implied explanation for this anomaly in the *Oxford English Dictionary* and in many books on post-truth reiterates a familiar binary of reason and emotion (e.g., d’Ancona, 2017; Kalpokas, 2019; McIntyre, 2018). The latter has replaced or suppressed the former, it is suggested, which, if it were true, would be a description more than an explanation. And this description is also an accusation against the epistemologically lapsed, those who let themselves be led astray by emotion in the shaping of their opinions, and the accusation is literally an ancient one. In the theory and practice of democracy — the regime in which public debate and political opinion is of paramount significance — the relationship between reason and emotion has always been troubled, the former recurrently seen as threatened or besieged by the latter. Responses to this predicament, from ancient Greece to our own day, have relied on the kind of argument that we are now again seeing in the discourse on post-truth: that reason must be rehabilitated and emotion checked (d’Ancona, 2017; McIntyre, 2018; Sim, 2019; Adorno, 1950; Aristotle, 1996; Dahl, 1989; Schumpeter, 1947; cf. Enroth, 2020b; Cartledge, 2016; Dunn, 1993; Ober, 1998; Ober, 2008). If this is what the fighting comes down to, then the new war on truth looks much like a war on reason waged by emotion, a timeless and endless pseudo-conflict in which the counter-attack can only be reason striking back, in self-defense, against emotion (Rosen, 1989: 4–5).

Reproducing, yet again, this ancient binary is of little help. Not only have political debate and public opinion always been shaped by what the *Oxford English Dictionary* refers to as “emotion and personal belief,” as much as by “objective facts” (cf. Achen & Bartels, 2016; Alexander, 2006; Enroth, 2020b), even leaving aside, for now, the immensely complex process through which objective facts are established and become compelling as such (cf. Daston & Galison, 2010; Latour, 2005). Moreover, this ancient tension between reason and emotion in the discourse of democracy is, in our current situation, a diversion, an inherited construct obscuring rather than elucidating what is presently going on. If sizeable sections of the populations of many democracies today respond to the emotional appeals of populist demagogues, to the point of enthusiastically embracing blatant lies, brazen deceit, and even violence, broaching this as reason being overtaken by emotion begs rather than answers the question of why and how this is happening, hence the need to change paradigms. Framing our predicament in epistemological terms — in terms of a loss of truth, facts, and reason — not only misses, but perilously misrepresents, what the predicament is and what we may and may not do about it.

## The War on the War on Truth

Instead of taking it at face value, we should read the epistemological diagnosis of our situation as itself symptomatic. As a point of entry, we may turn to the word “authority,” which occurs even in post-truth treatises that are firmly fixed in the epistemological paradigm. The word is typically unremarked, but it tends to appear at key junctures in the argument. “Post-truth was foreshadowed,” Lee McIntyre suggests, “by what happened to science over the last several decades.” What McIntyre believes happened to science over the last several decades was that it ceased to be “respected for the authority of its method,” and now finds its results “openly questioned by legions of nonexperts who happen to disagree with them. It is important to point out,” McIntyre continues, “that scientific results are routinely scrutinized by scientists themselves, but that is not what we are talking about here” (2018: 17).

Several things are noteworthy about these claims. For one, if we look beneath the surface manifestations of post-truth — that is, beneath epistemology — to its underlying dynamic, this is presented as a matter of authority, of the epistemic authority of science, while the nature of that authority remains unexplored. For another, this is presented as a case of authority in crisis, a crisis supposedly resulting from a certain kind of illicit questioning of authority. For yet another, it is suggested that the authority of science is intrinsically esoteric; “the authority of its method” appears as an arcanum to which nonexperts do not — and, notably, should not — have direct access, yet for which they should have respect. To question the authority of science can only be the prerogative of science itself; in this vision, the only admissable model for questioning science turns out to be peer-review, to which McIntyre makes explicit reference (2018: 17). On this telling, then, the story of post-truth is a story of undue exoteric questioning of the esoteric authority of science, with reason under the undue influence of emotion.

Compare and contrast this story with what amounts, at first blush, to an opposite position on the post-truth spectrum, a position most clearly stated and uncompromisingly adopted by Bruno Latour. What someone such as McIntyre finds deplorable in the post-truth situation, for Latour is a cause for celebration, even a *cause célèbre*. Science can no longer, Latour argues, without trying to curb his enthusiasm, maintain the “lofty and disinterested epistemology” on which its authoritative commands have traditionally rested, and we are all better off without it. This is most clearly illustrated, Latour suggests, by the issue of anthropogenic climate change, where the authority of science is being insistently contested by a range of interested parties (Latour, 2018: 79–80). Any “matters of fact” presented by science in this area will also be “matters of concern” for anyone with a stake in the matters at hand (Latour, 2017: 164).

If the traditional lofty and disinterested epistemology of science was always a chimera, and if the insistent questioning of climate science by nonexperts serves the purpose — which has long been Latour’s own express purpose in his theoretical work (Latour, 1993, 2004, 2005) — of highlighting this chimera as such, then the way forward can hardly be the way back. “With no hope of escaping the controversies” in which they now find themselves involved, scientists “would do better to organize themselves in order to resist all those that do take an interest – a great interest – in them” (Latour, 2018: 80). On this view, what is going on in the post-truth situation is not that science is inadmissably questioned by nonexperts, but that science and its critics have become combatants in “an acknowledged state of war” (Latour, 2017: 246), where “the smallest study will immediately be plunged into a full-scale battle of interpretations” (Latour, 2018: 79; cf. Latour, 2017: 245–246). In Latour’s reading of this situation, what science can hope to bring to the battle field is not the lofty and distinterested epistemology through which matters of fact are purportedly discovered and studied, but the power to enforce its own matters of concern on its adversaries, and on the Earth, on the model of Carl Schmitt’s *Nomos der Erde* (Latour, 2017: 220–254; Latour, 2018: 79; cf. Schmitt, 1996; Schmitt, 2003). Conjuring “violent disputes over the exegesis of scientific literature,” Latour envisions − and hopes for − a situation in which “the scientists are on the warpath” (2017: 228).

Ironically, this belligerent vision and the metaphorics of war and battle through which it is conveyed capture a central but unacknowledged aspect of the epistemological case against post-truth and how that case has been pursued. If Latour wants scientists on the warpath, those who battle post-truth by epistemology are in effect fighting a war by proxy, engaging in overt and covert attacks on familiar enemies in the American culture wars, with which, it turns out, the war on truth shares a vital battle front. It is not only post-truth which “flourishes amidst ‘culture wars’ and ideological polarization” (Newman, 2019: 98), as is often noted in the post-truth literature; the same is true of the fight against post-truth. Notably, and predictably, the insistent boogeyman called “postmodernism” has again raised its head. “Did postmodernism lead to post-truth?” McIntyre asks in a chapter title, rhetorically, since he immediately posits that “one of the saddest roots of the post-truth phenomenon seems to have come directly out of colleges and universities” (McIntyre, 2018: 123), a charge leveled, *inter alia*, against Latour (McIntyre, 2018: 141–143). “Think of this as the first thesis of postmodernism,” McIntyre implores his readers: “there is no such thing as objective truth.” The “second thesis of postmodernism,” according to McIntyre, one he ascribes to Foucault, or at least associates with his name, says that “any profession of truth is nothing more than a reflection of the political ideology of the person making it” (2018: 126).

Foucault famously wanted no truck with the concept of ideology, and the most cursory reading of his work makes it clear that the power he conceptualized and studied is not the kind of power a person may readily wield over others (Foucault, 1972; Foucault, 1984; cf. Hacking, 2002). But these are finer points that are not allowed to stand in the way of the strategy of the epistemologists in this battle: “to stake new ground in old domestic wars,” as Joan Didion put it in a different yet oddly similar context. That remark was made after “the death of postmodernism” had been declared, and celebrated, as a propitious side effect of the 9/11 attacks (Didion, 2003: 12). “It seemed bizarre that events so serious would be linked causally with a rarified form of academic talk,” Didion quoted Stanley Fish commenting at the time (Didion, 2003: 11). The same can be said today of the latest resurrection of this boogeyman in the current discourse on post-truth. The habitual postmodernist-bashing illustrates the local or parochial context in which post-truth discourse has emerged, and also what is at stake in the epistemological critique of post-truth. Weaponizing epistemology may — at least in the eyes of those who adopt this strategy — serve its purpose against established enemies in and out of the ivory tower, but this strategy says more about the particular cultural context in which it is adopted than it does about the pervasive underlying condition that has led to the post-truth misdiagnosis.

If the authority of science is waning, this should make us ask questions about authority, not epistemology. The positions I have reviewed here only get us so far. Epistemic authority cannot be reduced to the issuing of commands by experts in the name of truth, to be passively complied with by nonexperts. Nor can it be reduced to whatever power scientists may or may not be able to mobilize in a purported state of war (Enroth, 2020a). For want of an account of how authority is constituted and sustained, there is little of substance to say about the dynamic behind its waning, or the prospect of its reconstitution, except to urge scientists to go on the warpath, or offer such homelies as “one must always fight back against lies,” and “to fight back against post-truth is to fight it within ourselves” (McIntyre, 2018: 155, 162). The politico-theoretically oriented contributions to the post-truth literature likewise tend to the generic when it comes to this dynamic and the possibility of restoring what has been lost. In this regard, we are referred to “acting and judging politically” (Zerilli, 2019: 162), and to “the limiting and enabling material environment that hosts democratic politics” (Hyvönen, 2018: 48).

## Arendt on Authority

To make headway, we need to change our concepts and our order of business, starting with authority and working our way from there to notions of truth and facts rather than the reverse. And we need to leave the battlefield, metaphorically and de facto, and also the seminar room. As Arendt knew, authority is reducible neither to reasoned argument, nor to power. “Where arguments are used,” she suggested, “authority is left in abeyance.” And “where force is used, authority itself has failed” (1993b: 93). She also remarked that while there is no shortage of argument and power in our lives, “a constant, ever-widening and deepening crisis of authority has accompanied the development of the modern world in our century,” a crisis she took to be “political in origin and nature” (1993b: 91).

Much in Arendt is idiosyncratic, but her analysis of what authority can be and how it can be understood in modernity is very much to the point in our current situation. Transcending epistemological as well as politico-theoretical diagnoses of post-truth, Arendt’s musings on authority let us explore the ties that bind the epistemological to the political by letting us explore the ties that do or do not bind when authority is claimed or assumed. What we moderns have lost, according to Arendt, is “a very specific form” of authority, “resting on a foundation in the past as its unshaken cornerstone,” a foundation that “gave the world the permanence and durability which human beings need precisely because they are mortals – the most unstable and futile beings we know of.” The modern loss of this form of authority, Arendt explains, “is tantamount to the loss of the groundwork of the world” (1993b: 92, 95). For “a very specific form” of authority, this is no small thing. Arendt’s ruminations on authority play variations on the time-honored theme of modernity as loss, specifically as loss of foundations, a theme that is coeval with modernity itself (Alexander, 2013; Foucault, 1984; Rosen, 1989). In contrast to Arendt, I do not believe this crisis of authority to be constant or static, but intermittent and dynamic: a potential for crisis inherent in the dynamics of authority in modernity, actualized under certain conditions that I shall attempt to summarize and exemplify with an eye to our current situation. The post-truth predicament, I argue, is symptomatic and emblematic of those conditions.

I turn now to Arendt’s words on authority, which I will consider in some detail, before drawing out the implications for our understanding of post-truth. In Arendt’s account, authority begins — before it even is, or when it has yet to become, authority — with power, and, notably, with power conceived not as power over others, but as power *with* others, coterminous with what Arendt liked to call acting in concert (1958: 244). By reference to some of her own favored authorities, the “men of the American Revolution,” Arendt suggests that, to those men, “power came into being when and where people would get together and bind themselves through promises, covenants, and mutual pledges; only such power, which rested on reciprocity and mutuality, was real power and legitimate” (Arendt, 2006: 173). Note the conventional *differentia specifica* of authority — “legitimate” — making an appearance at this point, but power is not yet authority and authority is not power, Arendt repeatedly points out, especially against those who think of power as power over others (1993b: 93; 2006: 169–170, 174–176). Yet, she also suggests, authority nevertheless begins with — both arises from and refers back to — this foundational form of power.

Arendt makes this point by reference to her other favored authorities, the Romans. On the subject of the founding of the city of Rome and the territorial expansion of the Roman empire, she notes that the Romans “were bound to the specific locality of this one city,” yet they were also “capable of adding to the original foundation” of their city until “the whole of Italy and, eventually, the whole of the Western world” had been incorporated, “as though the whole world were nothing but Roman hinterland” (1993b: 120). This “adding to the original foundation,” Arendt calls “authority,” adding that the Latin word *auctoritas* “derives from the verb *augere*, ‘augment’, and what authority or those in authority constantly augment is the foundation” (1993b: 121–122). For the Romans, in Arendt’s interpretation, “all authority derives from this foundation, binding every act back to the sacred beginning of Roman history” (1993b: 123). “The authority of the living was always derivative,” Arendt concludes, depending “upon the authority of the founders, who no longer were among the living” (1993b: 122).

At this point, a certain inconsistency or tension presents itself in Arendt’s account of authority, since the authority of the founders is in fact not yet, at the founding, authority, but power, as Arendt herself points out in her discussion of the American experience of founding: power by way of promises, covenants, and mutual pledges. And if it is only in and by adding to the original foundation that the foundation becomes authoritative, does this not suggest that the authority of the founders is just as derivative of the authority — or the power – of the living as the reverse? Authority augments the foundation, Arendt suggests, but she also suggests that the foundation becomes authoritative only by being augmented. The promises, covenants, and mutual pledges through which the founders bound themselves to each other can become authoritative for those who follow their lead — that is, turned from power to authority — only retroactively, after the fact, only if and insofar as those who follow will in turn bind themselves to the founders by the same means (Enroth, 2020a).

This inconsistency or tension is arguably not Arendt’s, or rather, this is Arendt’s version of an inconsistency or tension that is inherent in the concept of authority in a democratic setting (McMahon, 1994; cf. Furedi, 2013: 406): authority is supposed to be binding, yet it becomes binding — which is to say, authoritative — only if those for whom it is supposed to be binding make it so by binding themselves to it. This is an aspect of the logical lacuna that Jacques Derrida has referred to as “the mystical foundation of authority” (1992). The idea of legitimacy standardly invoked to identify and individuate authority puts a label on, but does not explicate, this mystery (Connolly, 1983: 108; McMahon, 1994; Raz, 1979; cf. Enroth, 2013). Arendt’s account of authority makes the mystery somewhat less mystical, in that she is explicit about how authority, when successful, ties human beings together in space and time, not just vertically, in imposed relationships of command and compliance, but also horizontally, in the making and keeping of promises, and not just synchronically, in the present, but also diachronically, tying the present to the past and to the future. “The way the beginner starts whatever he intends to do lays down the law of action for those who have joined him in order to partake in the enterprise and to bring about its accomplishment,” Arendt remarked on the American Revolution and its authoritative legacy (2006: 205).

When successful on these terms, authority ties those who follow to those who claim or assume authority, which is also to tie those who follow authority to each other, by way of their authorities. To follow authority, on this view, is not to blindly or reflexively submit but to join those who claim authority in order to partake in their enterprise, which is how their claims to authority become authoritative. When in crisis, authority no longer ties us together in this sense, or ties only some of us together and, therefore, divide those who do from those who do not follow those who claim or assume authority. Arendt’s account of authority offers — largely thanks to the inconsistency or tension at the heart of it — important insights into the dynamic by which this binding does or does not occur. Centered on promises as an aspirational way of binding people together in lieu of any unshaken foundation, it points to what we may think of as a communal aspect of authority. This is one of the major advances of Arendt’s view of authority over its more familiar rivals: this is an aspect of authority that is typically ignored or rendered in terms too thin in standard juridico-political theories of authority centered on the notion of legitimacy, and essentialized and rendered in terms too thick in theories with implicit or explicit authoritarian tendencies (Furedi, 2013; Villa, 1996: 157–158).

Arendt’s account of authority highlights that the ties that bind us are not simply or solely commands with which we are compelled to comply, but promises that we ourselves render authoritative after the fact, by binding ourselves to each other by way of our authorities, thus repeating the promises from which authority originates. When authority fails, the failure is not just that commands are no longer complied with, but that commands are no longer complied with because the ties that bind us to each other in space and time have come undone. And when such ties have come undone, what has been lost is not just command and compliance, but the sense that we ourselves partake in the enterprise, whatever the enterprise might be, and that its accomplishment is, if not necessarily within reach, at least a possibility. And what has appeared instead is a looming sense that promises once made have not been kept, or that the promises made have been made not just by someone else, but for someone else.

## Crisis of Authority

So how does this Arendtian perspective on authority and its crisis help us understand the post-truth situation? As Arendt noted on the subject of truth, “the compelling force of factual truth is limited; it does not reach those who, not having been witnesses, have to rely on the testimony of others, whom one may or may not believe” (1978: 59). Contrary to popular misconception, I suggest that the post-truth situation is not about disbelief in facts and the decline of truth per se, nor is it about reason being overtaken by emotion, still less is it about the words, deeds and misdeeds of notorious liers and their enablers, however corrosive these may be. Rather, this situation is about generalized and intensified disbelief in the others on whom we all inevitably rely for testimony, in lieu of having been witnesses. And we are now in a position to see that this not-so-compelling force of factual truth is homogeneous with the ties that do or do not bind those who follow to those who claim or assume authority in what Arendt liked to call the realm of human affairs. These ties are no stronger or weaker than the aspirational promises from which authority originates, and this is as true of our allegiance to societal, political, and cultural institutions as it is of our belief in those on whose testimony we rely to arrive at factual truth.

If authority begins — and ends — with the making of promises, we may recall at this point some of the promises on which postwar figurations of authority have been predicated, and the enterprises in which we have been invited to partake. Consider, in the USA, the promise of upward social mobility that we have come to know as the American Dream, and the emphasis on work as the means to that end (cf. Shklar, 1991: 67), or consider the historically bequeathed promise of a federal government keeping its hands to itself, in the name of liberty (Campbell, 2014), or the promise of equality pursued by the civil rights movement and other emancipatory movements in the postwar period (Alexander, 2006). Consider, in certain parts of Western Europe, the promise that welfare state policies would equalize life chances in good times and take the force of the blow in bad times (Esping-Andersen, 1990; Kildal & Kuhnle, 2007; Rothstein, 1998).^1^ Consider, in Eastern Europe, the promise that the fall of the Soviet Union would usher in a brave new world of prosperity and democracy (Bohle & Greskovits, 2012; Kopechek & Wcislik, 2015). And then consider how these promises have fared, and whether and how we have been able to effectively partake in the enterprises shaped by them. Such promises, when kept, or seemingly kept, forged allegiances that became and remained, for a certain period of time, authoritative, ties that bound us, for better and for worse, to each other, by way of our authorities. When manifestly or ostensibly no longer kept, the promises still remain — if only as reminiscences of more promising times — but the ties break and the allegiances dissipate (Alexander, 2016; Alexander et al., 2021; Campbell, 2014; Piketty, 2015; cf. Enroth & Henriksson, 2019, 2021).

This is obviously not an exhaustive list of all relevant phenomena in the names of which authority has been claimed or assumed in the postwar period, nor could anyone hope to provide such a list. Authority is a notoriously complex phenomenon and a frustratingly slippery concept (Enroth, 2013; Furedi, 2013). I certainly do not suggest that my remarks here in any way provide theoretical saturation; I do not believe in the possibility — let alone the desirability — of a unified theory of authority. But I do believe that these are illustrative and instructive examples of the kind of grand, foundational promises on which our established forms of authority have been premised. It may well be in the nature of such grand, foundational promises that they can only ever be partially kept, in the sense of being recognized and institutionalized in politics, economy, law, and culture; their being aspirational may even be part of their compelling force (cf. Alexander, 2006). And to say that such promises can only ever be partially kept is to say that they are always bound to be partially broken (Alexander, 2006: 6). But when promises made are seen to diverge spectacularly from what we can in fact witness in our lives, they cease to be authoritative, that is, binding.

This, I suggest, is the big picture, the general background, from which the post-truth situation is a cut-out and a blow-up. And if that is so, then what we are seeing today is not so much that “objective facts are less influential in shaping public opinion than appeals to emotion and personal belief” (OED; McIntyre, 2018: 5), let alone that “truth has been eclipsed” (McIntyre, 2018: 5), as we are seeing an increasing disconnect between what people can bear witness to in their own lives and what they take to be recognized and institutionalized in politics, economy, law, and culture. The promised goods — social mobility, liberty, equality of life chances, prosperity, and democracy — can be aspirational only to an extent, only for so long and only for so many, before the promises lose their compelling force.

In consequence of such disconnect, that is, in the absence of ties that bind, authority is easily reduced by the disenchanted and their nominal spokespersons to mere power (cf. Arendt, 2006: 92–93), to the institutions in which authority has been vested, and those who speak and act in the name of those institutions are easily reduced to elites and experts exercising their power at a remove from, and at the expense of, the disenchanted (Enroth, 2020b; Müller, 2016; Kaltwasser et al., 2017). At this point, as Arendt knew, authority has already failed (1993b: 93). This is where populist parties and leaders today both supply for a demand for this message among the disenchanted and produce and reproduce the demand for which they supply (Mudde, 2007; Mudde & Kaltwasser, 2017). At the same time, populists make promises of their own, claiming or assuming the authority to speak and act in the name of the true people, in the very same breath as they reduce established forms of authority to the illegitimate power of elites (cf. Müller, 2016). What will come out of this is too soon to say, but all the while the general, poorly understood crisis of authority that I have tried to briefly diagnose here is exacerbated. One consequence of such a crisis, a consequence of which Arendt was keenly aware, is loss of the ability to distinguish between the authoritative and the authoritarian (1993b: 92). This goes some way towards explaining the lure of strongmen dressed up as populists, and the compelling force of the promises they make, for those for whom the ties of democratic authority no longer bind (Ben-Ghiat, 2020; Levitsky & Ziblatt, 2018; Levitsky & Way, 2020).

Most notably for the purposes of this piece, this is also how a reduction of authority to power, to institutions and elites at a remove, has engendered the specific manifestations of crisis on which the epistemological critique of post-truth is myopically focused. Starting from foundational promises that have come to seem broken to those who used to believe in them, it is but a short step from disbelief in those who speak and act in the name of established institutions to disbelief in that of which they speak — anthropogenic climate change, the corona virus pandemic, or the benefits of vaccines, say — or, for that matter, to belief in that of which they do not speak; for instance, that one’s political opponents are part of a satanistic pedophile cabal run out of the basement of a Washington pizzeria (Roose, 2020; cf. Fenster, 2008: 281). That this step is epistemologically considerable only illustrates the inadequacy of an epistemological diagnosis of and response to a political ailment.

## Reconstituting Authority

So what to do? If post-truth is a symptom rather than the ailment, then the first thing to do is to get the diagnosis right. When the sense that we ourselves partake in the enterprise — whatever the enterprise may be — is replaced by the sense that promises have been broken or made for someone else, the ensuing crisis can be expected to spread, metastasize, from the societal, the political, and the cultural to the epistemological, that is, to disbelief in the others on whose testimony we rely for the truth of phenomena such as anthropogenic climate change, the corona virus pandemic, and the benefits of vaccines. The spread is no doubt facilitated by demagogues, their enablers, and the algorithms on which our social media platforms run, but the potential for metastasis is inherent in the ailment. And if the diagnosis I have sketched is correct, then snappy slogans about scientists on the warpath and fighting back against lies will not cut it. Nor, as recent experiences surely illustrate, will “a greater emphasis on fact checking” (Sim, 2019: 156). A political ailment requires a political remedy (cf. Brooks, 2020).

Once this has been realized, and once it has been realized what this implies, what is needed is nothing less than a reconstitution of authority, which is to say, a compelling and tangible reconnection with the foundational promises on which established forms of authority rest, taking the measures needed for those promises to be recognized and institutionalized in politics, economy, law, and culture, or at least for the divergence between the promises on which authority rests and the institutions in which it is vested to be somewhat less spectacular. Clearly, this is no quick or easy fix. This would be a rebooting of our historically formed and transformed configurations of authority and community. In the specific, this remedy would be as variable and complex as the contexts in which authority is now in crisis. In practical and political terms, this may be inconvenient, but nothing is gained by a convenient remedy for a misdiagnosed illness.

In theoretical terms, this brings us back to the tension in the concept of authority in a democratic setting: when successful, authority is binding, yet it becomes binding only if those for whom it is supposed to be binding make it so by binding themselves to it. Arendt’s tracing of authority back to the power of foundational promises serves to remind us of this aspect of authority in modernity, and the ambiguity at the heart of her account serves to remind us of the always unfinished constitution of authority and community, which can only be a co-constitution between past and present, between those who claim authority and those who render authority authoritative by following those who claim it (Derrida, 1986; Honig, 1991; Enroth, 2020a). And such co-constitution is anything but a foregone conclusion. Arendt’s analysis of authority is an object lesson in the fragility of a common world.

This also brings us back to the vexed question of the questioning of authority. The critical spirit of modernity impels us to restlessly question authority (Koselleck, 1988). But questioning is not the same as dismissal or disbelief, which is what we are witnessing in the post-truth situation, nor is it the same as warfare — a reckless metaphor, even in social theory, a metaphor which, at best, reflects and reproduces rather than illuminates the polarization which is part of our predicament, and which, at worst, easily turns literal, as recent events have reminded us. The current crisis of authority stems not from an excess of questioning, as the epistemological critique of post-truth would have it, but from disbelief in — which is to say, loss of — authority. In Arendt’s terms, but not in her spirit, to question authority is itself to augment it, to symbolically sustain it through the very act of subjecting it to critique (cf. Alexander, 2006; Bartelson, 2001). Yet, if the foundational promises on which established forms of authority rest have come to seem farther removed from what people can bear witness to in their lives, at the same time, the conditions for questioning authority seem to have deteriorated (cf. Newman, 2019: 99–100). On both sides of the Atlantic, the notion that no real or viable choice exists in matters of policy or ideology — a notion popular with political and administrative elites, as well as with aspiring autocrats — has achieved just that: to pave the way for disbelief in authority by making authority seem unquestionable. And as Arendt also knew, when authority appears unquestionable, it degenerates either into anti-political authoritarianism or into apolitical administration (Arendt, 1993b) — that is, what we now know as “governance” (Enroth, 2014; Offe, 2009).

Loss of authority, Arendt once ventured, “does not entail, at least not necessarily, the loss of the human capacity for building, preserving, and caring for a world that can survive us and remain a place fit to live in for those who come after us” (1993b: 95). It is far from clear whether our current situation lends itself even to this moderate amount of optimism, but that wager may be our best bet.

## Footnotes

1 Needless to say, this is not to make a specific observation about different welfare regimes, only to note that, regardless of regime type, the welfare state evolved in response to “the social question” (Enroth & Henriksson, 2019), thus offsetting poverty even when and where active redistribution of wealth was not the objective.
